# Longitudinal CT-derived muscle–fat–bone remodeling following androgen deprivation therapy initiation in prostate cancer

**DOI:** 10.3389/fruro.2026.1985221

**Published:** 2026-10-08

**Authors:** Manuela Montatore, Gianmichele Muscatella, Federica Masino, Eluisa Muscogiuri, Giuseppe Guglielmi

**Affiliations:** 1 Department of Clinical and Experimental Medicine, Foggia University School of Medicine, Foggia, Italy; 2 Radiology Unit, “V. Emanuele II” Hospital, Bisceglie, Italy; 3 Radiology Unit, "A. Perrino" Hospital, Brindisi, Italy; 4 Radiology Unit, “M. Dimiccoli” Hospital, Barletta, Italy

**Keywords:** adipose tissue, androgen deprivation therapy, body composition, computed tomography, opportunistic imaging, prostate cancer, skeletal muscle, vertebral attenuation

## Abstract

**Introduction:**

Androgen deprivation therapy (ADT) is a cornerstone of prostate cancer treatment and is commonly accompanied by longitudinal changes in muscle, adipose tissue, and skeletal health that may be poorly captured by conventional anthropometric measures. This study investigated whether routine oncologic computed tomography (CT) could provide an integrated longitudinal assessment of muscle, adipose tissue, and vertebral trabecular attenuation following ADT initiation.

**Methods:**

In this retrospective, single-center longitudinal study, 60 consecutive men with prostate cancer initiating ADT between January 2023 and May 2025 were screened at Antonio Perrino Hospital, Brindisi, Italy, with clinical follow-up available through July 2026. Forty patients with an eligible baseline CT obtained within 30 days before ADT initiation and a technically comparable follow-up CT at 12 ± 2 months were included. At L3, skeletal muscle index (SMI), bilateral psoas attenuation, subcutaneous adipose tissue index (SATI), and visceral adipose tissue index (VATI) were assessed. Trabecular vertebral attenuation was measured at L1–L3.

**Results:**

Mean SMI decreased from 48.4 ± 6.9 to 45.5 ± 6.8 cm²/m² (−6.0%; p<0.001), while mean psoas attenuation decreased from 41.0 ± 5.9 to 37.8 ± 6.0 HU (−7.8%; p<0.001). SATI increased by 11.6% (p<0.001), VATI by 6.2% (p=0.018), and mean vertebral trabecular attenuation decreased by 4.4% (p=0.006). BMI changed only modestly and showed a weak relationship with SMI change. Twenty-three patients (57.5%) had an SMI decline ≥5%, a prespecified exploratory threshold. In an exploratory parsimonious adjusted analysis, lower baseline psoas attenuation was associated with subsequent SMI decline ≥5%; however, the limited sample size precludes interpretation as an independent predictive biomarker.

**Discussion:**

Routine oncologic CT demonstrated concurrent longitudinal changes in muscle, adipose tissue, and vertebral attenuation following ADT initiation that were only weakly reflected by BMI. These findings support further evaluation of integrated opportunistic CT phenotyping in larger controlled cohorts.

## 1 Introduction

Prostate cancer is among the most commonly diagnosed malignancies in men worldwide and encompasses a broad clinical spectrum ranging from localized tumors to node-positive and metastatic disease (1). Androgen deprivation therapy (ADT) remains a fundamental component of treatment across several disease settings, including high-risk and locally advanced prostate cancer treated with radiotherapy and metastatic disease, increasingly within multimodal therapeutic strategies (2–4). As survival improves and exposure to systemic treatment becomes more prolonged, the clinical consequences of therapy beyond conventional tumor control have gained increasing relevance, particularly in an ageing population frequently affected by cardiovascular, metabolic, and musculoskeletal comorbidities.

The systemic effects of androgen suppression extend well beyond the prostate. ADT is associated with loss of lean and skeletal muscle mass, increased adiposity, metabolic alterations, and deterioration of skeletal health (5–7). These changes may develop simultaneously and are not necessarily reflected by conventional measures such as body weight or body mass index (BMI). An increase in adipose tissue may coexist with progressive muscle depletion, potentially masking clinically relevant tissue remodeling when assessment relies predominantly on weight-based measures (8). Moreover, muscle, adipose tissue, and bone should not be regarded as isolated compartments. Their biological interaction involves mechanical loading, hormonal signaling, inflammatory pathways, and metabolic crosstalk, providing a rationale for evaluating longitudinal changes across these compartments within an integrated imaging framework rather than considering each tissue component in isolation.

Body composition abnormalities in men with prostate cancer have increasingly been associated with clinically relevant outcomes. A systematic review and meta-analysis demonstrated associations between muscle and fat compartments and overall survival in prostate cancer, supporting the prognostic relevance of body composition beyond conventional anthropometry (9, 10). More specifically, Chiang et al. showed that skeletal muscle loss during ADT was associated with increased non-cancer mortality in patients with high-risk prostate cancer, whereas BMI did not provide comparable prognostic information (11). Subsequent longitudinal evidence identified treatment-related changes in skeletal muscle and adipose tissue as predictors of survival during ADT, with clinically meaningful changes occurring across multiple body compartments (12). More recently, longitudinal muscle loss and fat gain observed during ADT have also been associated with subsequent cardiovascular events, further suggesting that changes in body composition during treatment may reflect broader patient vulnerability beyond oncologic status alone (13, 14).

Computed tomography provides a particularly valuable opportunity to investigate these changes. CT examinations are routinely performed in patients with prostate cancer for staging, treatment assessment, and oncologic surveillance and therefore contain quantitative information that can be extracted without additional imaging acquisition. Cross-sectional analysis at the third lumbar vertebral level has become an established approach for estimating skeletal muscle and abdominal adipose tissue compartments in patients with cancer (15, 16). Beyond muscle quantity, CT attenuation can provide complementary information regarding muscle composition, while measurements of visceral and subcutaneous adipose tissue characterize fat distribution more accurately than BMI alone. Routine CT may also provide opportunistic information on skeletal status through measurement of vertebral trabecular attenuation. This parameter adds complementary information because skeletal integrity is not captured by muscle or adipose tissue measurements alone and is particularly relevant in men receiving long-term androgen suppression. Incorporating vertebral attenuation therefore extends body-composition analysis into a three-compartment muscle–fat–bone framework using the same routinely acquired examination. In men with prostate cancer, CT-derived measurements of muscle and bone have previously demonstrated associations with non-cancer mortality, further supporting the potential clinical value of information already embedded within standard oncologic imaging (17).

However, previous investigations have frequently focused on individual body-composition compartments, selected disease populations, or specific clinical outcomes. The incremental value of the present study lies in the simultaneous longitudinal assessment of skeletal muscle quantity, muscle attenuation, visceral and subcutaneous adiposity, and vertebral trabecular attenuation within the same routinely acquired oncologic CT examinations. This integrated framework allows concurrent muscle, fat, and skeletal changes to be evaluated within a single imaging dataset and compared with conventional BMI assessment. We therefore aimed to characterize longitudinal CT-derived changes in these compartments following ADT initiation and to determine whether they were adequately reflected by BMI. Secondary objectives were to explore clinical factors associated with greater SMI decline and, in a hypothesis-generating analysis, subsequent clinical outcomes.

## 2 Materials and methods

### 2.1 Study design and population

This retrospective, single-center longitudinal observational study was conducted at Antonio Perrino Hospital, Brindisi, Italy. Consecutive men with histologically confirmed prostate cancer who initiated androgen deprivation therapy (ADT) between January 2023 and May 2025 were screened. Clinical and imaging follow-up was available through July 2026.

Patients were eligible if they had: (I) histologically confirmed prostate cancer; (II) a clearly documented date of ADT initiation; (III) an abdominal or abdominopelvic contrast-enhanced CT examination performed within 30 days before treatment initiation (T0); (IV) a follow-up CT examination performed 12 ± 2 months after ADT initiation (T1); (V) complete visualization of the L1–L3 vertebral levels and the entire abdominal musculature at L3; and (VI) sufficient clinical and anthropometric data to calculate normalized body composition indices.

Sixty consecutive patients were initially screened. Twenty were excluded: seven because no eligible baseline CT was available, six because no follow-up CT was performed within the predefined 10–14-month interval, three because they died before an eligible follow-up CT could be obtained, two because paired CT examinations were technically unsuitable for reliable longitudinal quantitative analysis, and two because essential anthropometric or clinical data were unavailable. The final longitudinal cohort therefore comprised 40 patients (**Figure 1**).

**Figure 1:**
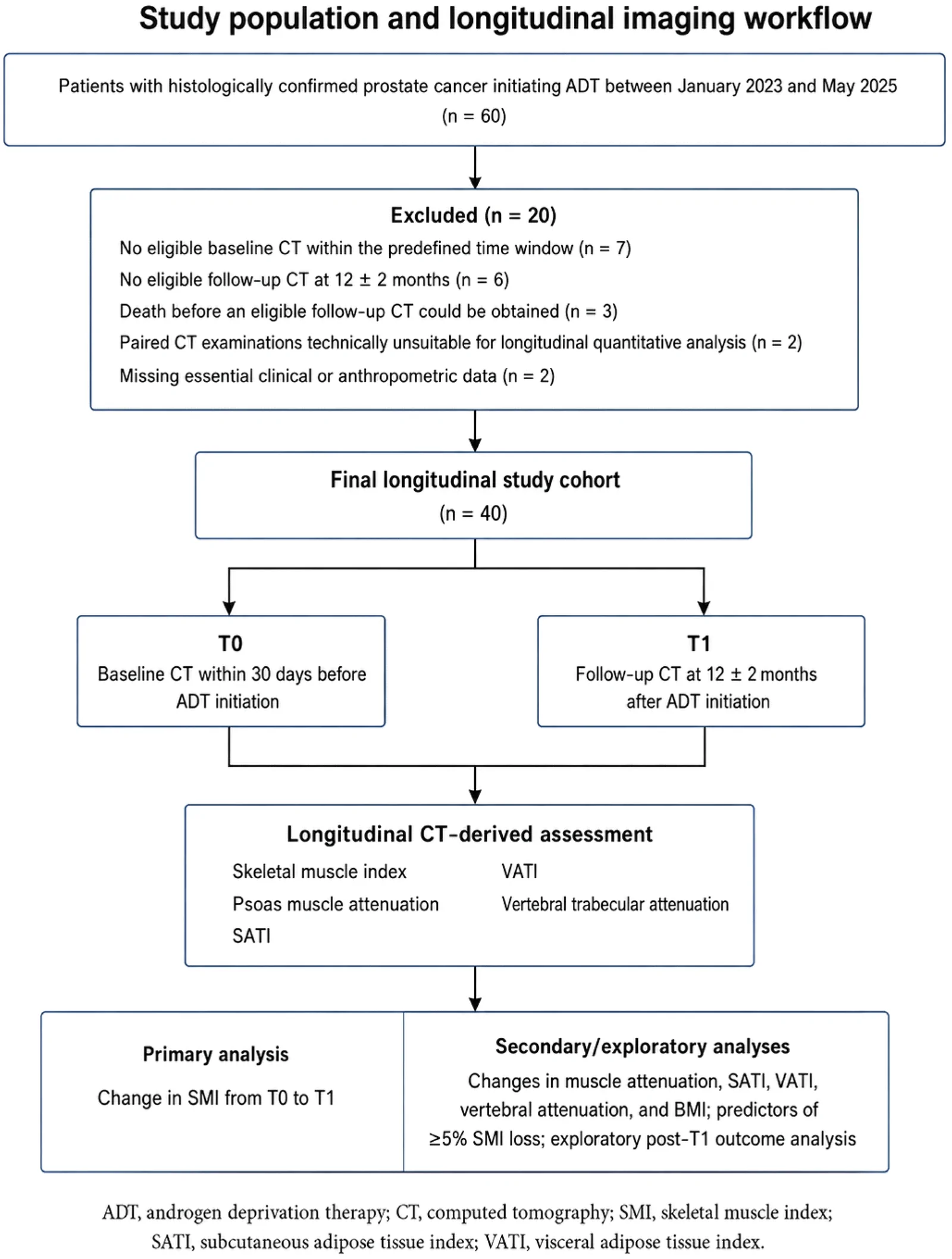
Study population and longitudinal imaging workflow. Flow diagram illustrating patient screening, reasons for exclusion, final cohort selection, and timing of CT-derived body composition assessment relative to androgen deprivation therapy. T0 was defined as the eligible CT examination obtained within 30 days before ADT initiation, whereas T1 was defined as the technically comparable follow-up examination obtained 12 ± 2 months after treatment initiation. ADT, androgen deprivation therapy; CT, computed tomography; SMI, skeletal muscle index; SATI, subcutaneous adipose tissue index; VATI, visceral adipose tissue index.

ADT included gonadotropin-releasing hormone agonists or antagonists. Additional androgen-receptor pathway inhibitors, chemotherapy, and radiotherapy were recorded as concomitant treatments rather than considered part of the ADT definition. Patients were not excluded on the basis of metastatic status, allowing inclusion of a real-world spectrum of locally advanced, node-positive, and metastatic disease. Baseline comorbidities, including hypertension, diabetes, cardiovascular disease, and chronic kidney disease, may also influence muscle composition, metabolic status, and physical activity. Their independent effects could not be reliably modeled in the present sample and therefore remain a potential source of residual confounding.

The study was conducted in accordance with the principles of the Declaration of Helsinki. Clinical and imaging data were retrospectively retrieved and anonymized before analysis. According to institutional requirements for retrospective research using anonymized data, the requirement for individual informed consent was waived.

### 2.2 Clinical and treatment variables

Demographic, anthropometric, oncologic, and treatment-related variables were retrieved from electronic medical records. Collected variables included age at ADT initiation, height, body weight at T0 and T1, and body mass index (BMI). Major baseline comorbidities included arterial hypertension, diabetes mellitus, established cardiovascular disease, and chronic kidney disease.

Oncologic variables included baseline prostate-specific antigen (PSA), histologic grade according to the available Gleason score and/or ISUP Grade Group, T stage, nodal status, metastatic status, presence and site of distant metastases, and treatment setting. Disease extent was recorded according to the available TNM classification at ADT initiation. ADT class and concomitant oncologic treatments, including androgen-receptor pathway inhibitors, chemotherapy, and radiotherapy, were also recorded. For variables not available in all patients, the number of patients with available data was reported.

BMI was calculated as body weight divided by height squared (kg/m²). Changes in BMI and all CT-derived quantitative parameters were calculated both as absolute differences and as percentage changes between T0 and T1.

### 2.3 CT acquisition and image selection

All CT examinations had originally been performed for routine oncologic indications, and no additional CT examination was obtained for study purposes. Quantitative analyses were restricted to contrast-enhanced portal venous phase images at both T0 and T1, thereby minimizing phase-dependent variability in attenuation measurements. Standardization of contrast phase is particularly relevant when longitudinal CT attenuation is used as a quantitative biomarker because enhancement phase can influence muscle radiodensity measurements (18).

The institutional oncologic CT protocol consisted of multidetector CT acquisition following intravenous administration of iodinated contrast material, with portal venous phase imaging obtained approximately 70–80 s after contrast administration. Images used for quantitative assessment were reconstructed with a slice thickness of ≤3 mm. Automated tube-current modulation was routinely used, while tube voltage was predominantly 120 kVp. For longitudinal comparison, paired examinations were required to have the same contrast phase and comparable acquisition and reconstruction parameters; marked discrepancies in tube voltage, reconstruction thickness, or image quality constituted grounds for exclusion from attenuation-based longitudinal analysis. This approach was chosen to prioritize within-patient technical comparability rather than pooling measurements obtained from different enhancement phases. Chiang et al. similarly performed longitudinal body composition assessment using standardized portal venous phase CT at 70 s, 120 kVp, and 3-mm reconstructed images (11).

For each patient, T0 was defined as the eligible CT examination closest to, but preceding, ADT initiation. The actual interval between the T0 CT examination and ADT initiation was recorded for each patient and summarized as median and interquartile range. T1 was defined as the eligible examination closest to 12 months after treatment initiation within the predefined 10–14-month window. When more than one examination fulfilled these criteria, the examination closest to the target time point was selected.

Because the study was retrospective, complete information on scanner model, contrast-agent dose, and reconstruction kernel was not uniformly available for all examinations. Technical matching therefore focused on contrast phase, tube voltage, reconstructed slice thickness, and overall image quality.

### 2.4 CT-derived skeletal muscle and adipose tissue assessment

Body composition analysis was performed on a single axial CT section at the level of the third lumbar vertebra (L3), using the same anatomical landmark at T0 and T1. The L3 level was selected because cross-sectional skeletal muscle and adipose tissue measurements at this level have been extensively validated as surrogates of whole-body tissue compartments in oncologic populations.

Images were analyzed using a dedicated workstation with semiautomated threshold-based segmentation and manual correction when required. The reader performing the primary measurements was blinded to subsequent clinical outcomes.

Total skeletal muscle segmentation included the bilateral psoas, paraspinal muscles, quadratus lumborum, rectus abdominis, transversus abdominis, and internal and external oblique muscles. Skeletal muscle tissue was identified using an attenuation range of −29 to +150 HU, consistent with established CT body composition methodology (15, 16). The total cross-sectional skeletal muscle area (SMA) was expressed in cm². Skeletal muscle index (SMI) was calculated as SMA divided by height squared and was expressed in cm²/m².

In addition to muscle quantity, muscle attenuation was assessed as a marker of muscle composition. Standardized regions of interest were placed within the right and left psoas muscles on the same L3 section, avoiding the muscular fascia, visible vessels, focal calcifications, and macroscopic intramuscular fat. The mean of the bilateral measurements was used as mean psoas attenuation (HU). Lower attenuation was interpreted as greater fatty infiltration of skeletal muscle rather than as a standalone diagnosis of sarcopenia. CT-derived muscle attenuation is considered complementary to muscle quantity because tissue radiodensity reflects characteristics not captured by cross-sectional area alone (19).

Subcutaneous adipose tissue (SAT) and visceral adipose tissue (VAT) were segmented separately at the same L3 level. SAT was defined as adipose tissue between the skin and the external boundary of the abdominal wall musculature, whereas VAT comprised adipose tissue located within the abdominal muscular compartment. Intermuscular adipose tissue was not included within either compartment.

Following the methodology used in longitudinal ADT studies, attenuation thresholds of −190 to −30 HU for SAT and −150 to −50 HU for VAT were applied, followed by manual correction of segmentation boundaries when necessary. SAT and VAT areas were expressed in cm². Subcutaneous adipose tissue index (SATI) and visceral adipose tissue index (VATI) were calculated by dividing the corresponding tissue area by height squared and were expressed in cm²/m². No population-derived diagnostic cut-off for visceral or subcutaneous adiposity was imposed. SATI and VATI were analyzed primarily as continuous longitudinal variables.

### 2.5 Vertebral trabecular attenuation

Opportunistic assessment of skeletal status was performed using vertebral trabecular attenuation at L1, L2, and L3. CT attenuation was recorded in Hounsfield units using an oval region of interest placed within the central trabecular compartment of each vertebral body. Cortical bone, endplates, the basivertebral venous plexus, focal sclerotic or lytic abnormalities, and areas affected by prominent artifact were avoided.

Vertebral levels affected by metastatic disease, compression fracture, vertebral augmentation, or other focal abnormalities potentially altering attenuation were excluded individually from bone analysis. Patients remained eligible for muscle and adipose tissue analyses when an individual vertebral level was unsuitable. Mean vertebral attenuation was calculated from the evaluable L1–L3 levels, with at least two technically suitable vertebrae required for longitudinal bone analysis. For each patient, only vertebral levels evaluable at both T0 and T1 were included, and the same matched vertebral levels were used to calculate mean attenuation at both time points. ROI placement and sampled area were reproduced as closely as technically possible at corresponding vertebral levels.

Because all bone measurements were obtained from contrast-enhanced CT, absolute vertebral attenuation values were not interpreted as equivalent to DXA-derived bone mineral density and were not used to diagnose osteoporosis. Instead, the within-patient percentage change in portal-phase trabecular attenuation between technically comparable T0 and T1 examinations was used as an opportunistic marker of longitudinal skeletal change. Previous work in men with prostate cancer supports the clinical relevance of CT-derived vertebral and muscle measurements obtained from routinely acquired imaging (17).

### 2.6 Study endpoints

The primary endpoint was the percentage change in SMI between T0 and T1. A ≥5% reduction in SMI was prespecified as an exploratory threshold for dichotomizing longitudinal SMI change in secondary analyses. Similar relative-change thresholds have been used in previous longitudinal oncologic and ADT studies (11, 12); however, this value was not considered a validated biological, diagnostic, or clinically established cut-off. The primary endpoint remained SMI change analyzed as a continuous percentage variable.

Clinical factors associated with SMI decline ≥5% were additionally explored as secondary hypothesis-generating analyses.

Because longitudinal SMI change could only be determined after acquisition of T1, any subsequent survival assessment required the T1 CT as the landmark time point. Patients were followed from T1 until death or the last available clinical follow-up. Given the very limited number of deaths anticipated in this cohort, survival analysis was prespecified as exploratory and hypothesis-generating and was not considered an inferential endpoint of the study.

### 2.7 Reproducibility analysis

To evaluate measurement reproducibility, 10 patients were randomly selected from the study cohort. Both baseline and follow-up CT examinations from these patients were independently reassessed by a second radiologist blinded to the initial measurements and clinical information.

Reproducibility was evaluated for SMI, mean psoas attenuation, SATI, VATI, and vertebral trabecular attenuation. Interobserver agreement was quantified using intraclass correlation coefficients (ICC). Intra-observer reproducibility was not separately assessed.

### 2.8 Statistical analysis

Continuous variables were reported as mean ± standard deviation when approximately normally distributed and as median with interquartile range otherwise. Categorical variables were summarized as numbers and percentages. Normality was assessed on the paired within-patient differences rather than on the T0 and T1 distributions separately.

Paired T0 and T1 measurements were compared using the paired Student’s t-test for normally distributed differences and the Wilcoxon signed-rank test otherwise. Absolute and percentage changes were reported where appropriate. Similar paired approaches have been used in previous longitudinal CT body composition studies during ADT (11, 12).

For paired comparisons analyzed using the paired Student’s t-test, mean within-patient differences and corresponding 95% confidence intervals were reported. For non-normally distributed paired differences analyzed using the Wilcoxon signed-rank test, an appropriate nonparametric estimate and confidence interval were reported where applicable.

For each patient, percentage change was calculated as [(T1 − T0)/T0] × 100. Cohort-level relative changes represent the mean of the individual percentage changes rather than the percentage difference between group means.

The relationship between percentage change in BMI and percentage change in SMI was assessed using Spearman’s rank correlation coefficient and displayed graphically using a scatterplot. This analysis was predefined to evaluate whether conventional weight-based assessment reflected CT-detected skeletal muscle change.

For exploratory secondary analyses, patients were classified according to SMI decline ≥5% versus <5%. An exploratory parsimonious logistic regression model was used to examine associations with SMI decline ≥5%. Given the limited cohort size and 23 patients meeting this threshold, the model was restricted *a priori* to three covariates: age, baseline psoas attenuation, and metastatic disease. The model was not intended as a comprehensive predictive model, and no automated or stepwise variable-selection procedure was used. Additional clinical and treatment-related covariates were not entered simultaneously because this would have increased the risk of model instability and overfitting.

For the exploratory survival analysis, Kaplan–Meier curves were generated from the T1 landmark and compared descriptively using the log-rank test according to SMI-decline group. Given the limited number of outcome events, this analysis was considered hypothesis-generating; no multivariable survival model was performed, and no conclusion regarding prognostic significance was prespecified.

Interobserver agreement was assessed using a two-way random-effects, absolute-agreement, single-measure intraclass correlation coefficient. The primary inferential endpoint was the longitudinal change in SMI. Given the exploratory nature of the secondary imaging and outcome analyses, no formal adjustment for multiple comparisons was applied; secondary p-values should therefore be interpreted as exploratory. All tests were two-sided, and a p-value <0.05 was considered statistically significant.

No formal *a priori* sample-size calculation was performed because of the retrospective design. All consecutive patients meeting the predefined eligibility criteria during the study period were included. Statistical analyses were performed using jamovi, version 2.7 (The jamovi project, Sydney, Australia).

## 3 Results

### 3.1 Study population

Sixty consecutive men with histologically confirmed prostate cancer were screened. Twenty patients were excluded according to the predefined eligibility criteria: seven had no eligible baseline CT, six had no follow-up CT within the 10–14-month interval, three died before an eligible follow-up CT could be obtained, two had paired CT examinations unsuitable for reliable longitudinal quantitative assessment, and two lacked essential clinical or anthropometric information. The final study cohort comprised 40 patients (**Figure 1**).

The median interval between the T0 CT examination and ADT initiation was 15.0 days [IQR 9.8–20.0]. Mean age at ADT initiation was 71.8 ± 6.9 years, and mean baseline BMI was 26.7 ± 3.5 kg/m². Twenty-five patients (62.5%) were overweight or obese. Baseline PSA was available in 38 patients, with a median value of 15.2 ng/mL [IQR 7.8–36.5]. Gleason score was available in 39 patients: 4/39 (10.3%) had a Gleason score ≤6, 16/39 (41.0%) had a score of 7, 9/39 (23.1%) had a score of 8, and 10/39 (25.6%) had a score of 9–10. Fifteen patients (37.5%) had T1–T2 disease, 20 (50.0%) had T3 disease, and 5 (12.5%) had T4 disease. Sixteen patients (40.0%) had node-positive disease and 14 (35.0%) had metastatic disease; bone metastases were present in 11 patients (27.5%) and visceral metastases in 3 (7.5%).

Twenty-two patients (55.0%) received a gonadotropin-releasing hormone agonist and 10 (25.0%) a gonadotropin-releasing hormone antagonist, while eight (20.0%) received another androgen-suppressive strategy within a combined systemic treatment regimen. Seventeen patients (42.5%) received a concomitant androgen-receptor pathway inhibitor, six (15.0%) received chemotherapy, and 18 (45.0%) received radiotherapy during the observation period. Major baseline comorbidities included arterial hypertension in 24 patients (60.0%), diabetes mellitus in 9 (22.5%), established cardiovascular disease in 8 (20.0%), and chronic kidney disease in 4 (10.0%). The median interval between ADT initiation and T1 CT was 12.1 months [IQR 11.4–12.8] (**Table 1**).

**Table 1:** Baseline clinical, oncologic, and treatment characteristics.

Characteristic	Overall cohort (n = 40)
Age, years	71.8 ± 6.9
BMI, kg/m²	26.7 ± 3.5
Overweight/obesity, n (%)	25 (62.5)
Baseline PSA, ng/mL	15.2 [7.8–36.5]
Baseline PSA available, n	38
Gleason score available, n	39
Gleason score ≤6, n/N (%)	4/39 (10.3)
Gleason score 7, n/N (%)	16/39 (41.0)
Gleason score 8, n/N (%)	9/39 (23.1)
Gleason score 9–10, n/N (%)	10/39 (25.6)
T1–T2 disease, n (%)	15 (37.5)
T3 disease, n (%)	20 (50.0)
T4 disease, n (%)	5 (12.5)
Node-positive disease, n (%)	16 (40.0)
Metastatic disease, n (%)	14 (35.0)
Bone metastases, n (%)	11 (27.5)
Visceral metastases, n (%)	3 (7.5)
GnRH agonist, n (%)	22 (55.0)
GnRH antagonist, n (%)	10 (25.0)
Other/combined androgen suppression, n (%)	8 (20.0)
Concomitant ARPI, n (%)	17 (42.5)
Chemotherapy, n (%)	6 (15.0)
Radiotherapy, n (%)	18 (45.0)
Arterial hypertension, n (%)	24 (60.0)
Diabetes mellitus, n (%)	9 (22.5)
Cardiovascular disease, n (%)	8 (20.0)
Chronic kidney disease, n (%)	4 (10.0)
T0 CT-to-ADT interval, days	15.0 [9.8–20.0]
ADT-to-T1 interval, months	12.1 [11.4–12.8]

Continuous variables are reported as mean ± standard deviation or median [interquartile range], as appropriate; categorical variables are reported as n (%). Percentages for variables with missing data were calculated using the number of patients with available information as the denominator. ADT, androgen deprivation therapy; ARPI, androgen-receptor pathway inhibitor; BMI, body mass index; GnRH, gonadotropin-releasing hormone; PSA, prostate-specific antigen.

### 3.2 Technical comparability and feasibility of vertebral attenuation analysis

#### 3.2.1 Technical comparability of CT examinations

All paired CT examinations included in the longitudinal quantitative analysis were acquired in the portal venous phase at both T0 and T1. Tube voltage was matched within each patient pair and was 120 kVp in 35/40 patients (87.5%) and 100 kVp in 5/40 (12.5%). Reconstructed slice thickness ranged from 2.5 to 3.0 mm in all examinations. No included CT pair showed relevant between-time-point differences in contrast phase, tube voltage, or reconstruction thickness that were considered sufficient to preclude attenuation-based comparison.

#### 3.2.2 Feasibility of vertebral analysis

Vertebral trabecular attenuation analysis was feasible in 111 of 120 potentially assessable vertebral levels (92.5%). Vertebral trabecular attenuation analysis included all 40 patients, with 111 matched vertebral levels available across T0 and T1. Complete L1–L3 assessment was available in 31 of 40 patients (77.5%), while the remaining 9 patients contributed two evaluable vertebral levels each. Excluded vertebral levels were omitted because of focal metastatic involvement (n = 5), marked degenerative change or compression deformity (n = 2), or image artifact/limited evaluability (n = 2). At least two evaluable vertebral levels were available in all patients included in the vertebral attenuation analysis (**Figure 2**).

**Figure 2:**
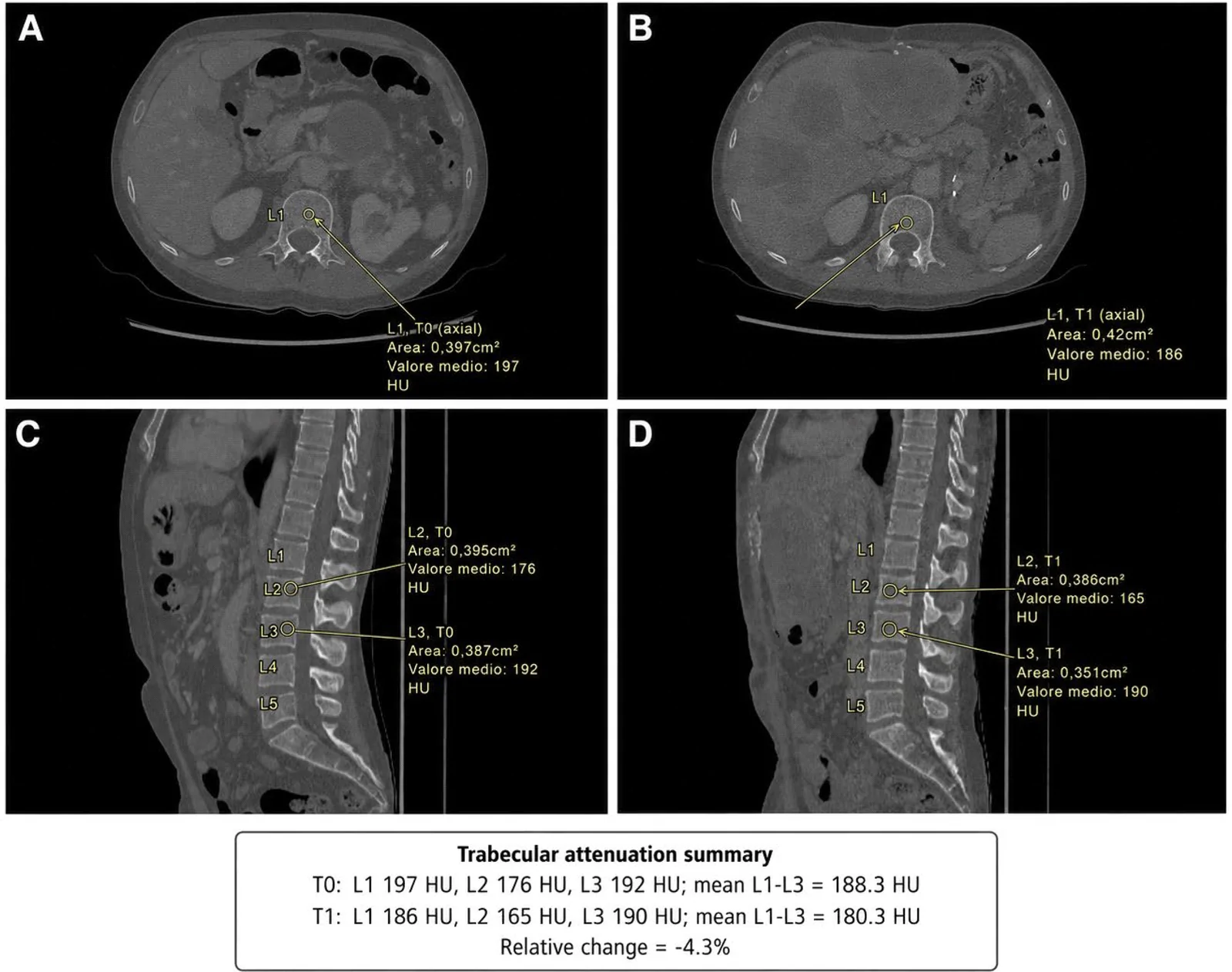
Representative example of longitudinal vertebral trabecular attenuation assessment on routine oncologic CT. Baseline [**(A, C)**; T0] and follow-up [**(B, D)**; T1] portal venous phase CT images from the same patient are shown in the axial and sagittal planes. Vertebral trabecular attenuation was measured at L1, L2, and L3 using regions of interest placed within the central cancellous portion of each vertebral body, avoiding cortical bone, endplates, focal osseous lesions, and visible artifacts. Mean L1–L3 attenuation decreased from 188.3 HU at T0 to 180.3 HU at T1, corresponding to a relative reduction of 4.3%. CT, computed tomography; HU, Hounsfield units; ROI, region of interest; T0, baseline; T1, follow-up.

### 3.3 Longitudinal CT-derived changes following ADT initiation

The primary endpoint, longitudinal change in SMI, showed a significant mean reduction between T0 and T1. Mean SMI decreased from 48.4 ± 6.9 to 45.5 ± 6.8 cm²/m², corresponding to a mean individual relative reduction of 6.0% and a mean paired difference of −2.90 cm²/m² (95% CI −3.96 to −1.84; p<0.001). Twenty-three patients (57.5%) had an SMI decline ≥5%.

Mean bilateral psoas attenuation decreased from 41.0 ± 5.9 to 37.8 ± 6.0 HU, with a mean paired difference of −3.20 HU (95% CI −4.35 to −2.05; p<0.001). SATI increased from 52.5 ± 17.4 to 58.6 ± 18.8 cm²/m², corresponding to a mean paired difference of +6.10 cm²/m² (95% CI + 3.54 to +8.66; p<0.001), whereas VATI increased from 50.1 ± 18.3 to 53.2 ± 19.1 cm²/m², with a mean paired difference of +3.10 cm²/m² (95% CI + 0.56 to +5.64; p=0.018). Mean vertebral trabecular attenuation decreased from 132.1 ± 24.6 to 126.3 ± 24.1 HU, corresponding to a mean paired difference of −5.80 HU (95% CI −9.82 to −1.78; p=0.006). In contrast, BMI changed only modestly, from 26.7 ± 3.5 to 27.0 ± 3.6 kg/m², with a mean paired difference of +0.30 kg/m² (95% CI −0.18 to +0.78; p=0.21) (**Table 2**).

**Table 2:** Longitudinal anthropometric and CT-derived changes following ADT initiation.

Parameter	T0	T1	Mean individual relative change	Mean paired change (95% CI)	p-value
BMI, kg/m²	26.7 ± 3.5	27.0 ± 3.6	+1.1%	+0.30 (−0.18 to +0.78)	0.21
SMI, cm²/m²	48.4 ± 6.9	45.5 ± 6.8	−6.0%	−2.90 (−3.96 to −1.84)	<0.001
Psoas attenuation, HU	41.0 ± 5.9	37.8 ± 6.0	−7.8%	−3.20 (−4.35 to −2.05)	<0.001
SATI, cm²/m²	52.5 ± 17.4	58.6 ± 18.8	+11.6%	+6.10 (+3.54 to +8.66)	<0.001
VATI, cm²/m²	50.1 ± 18.3	53.2 ± 19.1	+6.2%	+3.10 (+0.56 to +5.64)	0.018
Vertebral attenuation, HU	132.1 ± 24.6	126.3 ± 24.1	−4.4%	−5.80 (−9.82 to −1.78)	0.006

Representative examples of longitudinal vertebral attenuation and body composition assessment are shown in **Figures 2**, **3**. Individual T0-to-T1 trajectories for the major anthropometric and CT-derived parameters are shown in **Supplementary Figure S2**, illustrating the interindividual heterogeneity of longitudinal change across muscle, adipose, and vertebral compartments. Values at T0 and T1 are reported as mean ± standard deviation. Relative changes represent the mean of individual within-patient percentage changes. Paired changes are expressed as T1 minus T0 with corresponding 95% confidence intervals. BMI, body mass index; CT, computed tomography; HU, Hounsfield units; SATI, subcutaneous adipose tissue index; SMI, skeletal muscle index; VATI, visceral adipose tissue index.

**Figure 3:**
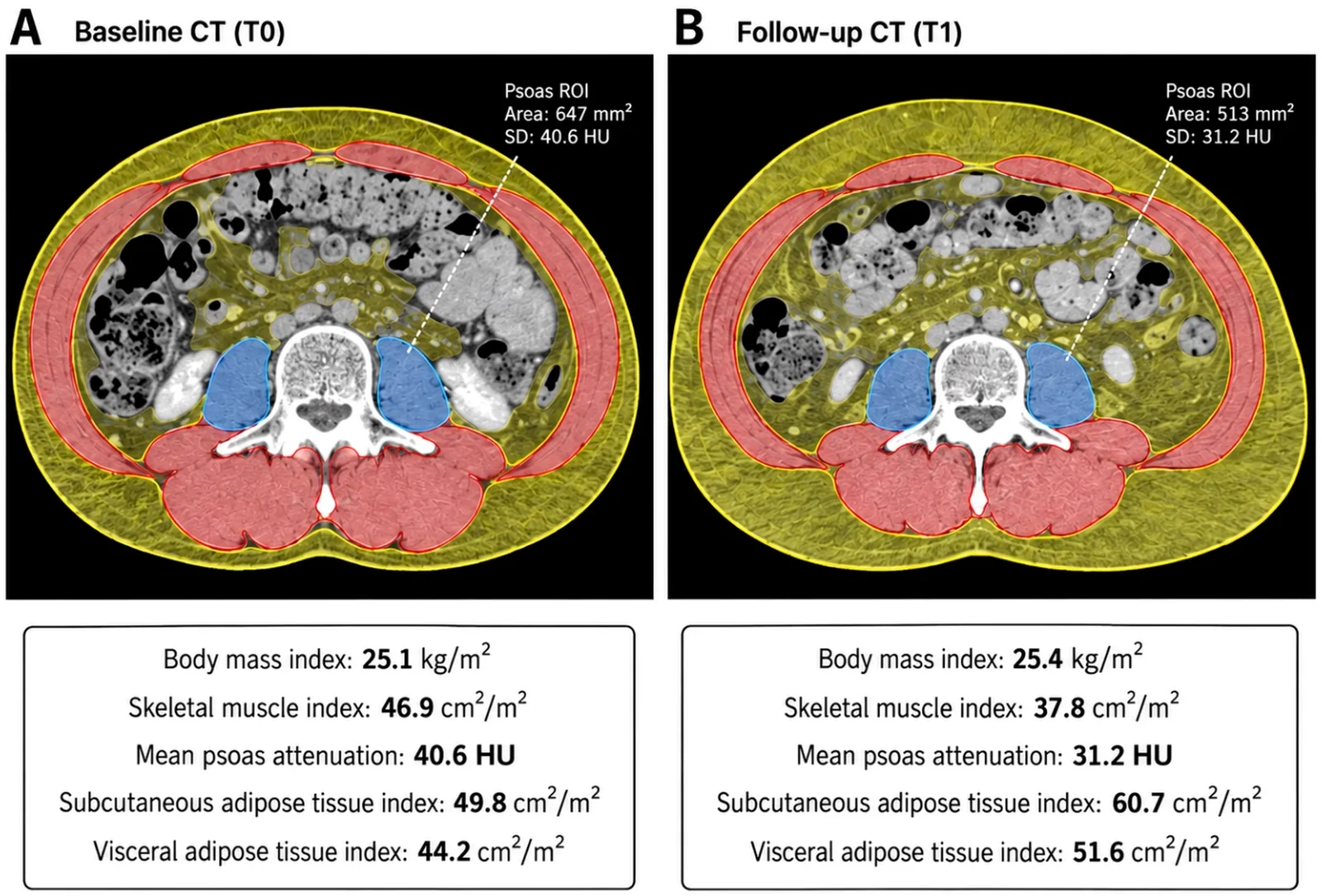
Representative longitudinal CT-derived body-composition assessment at the L3 level. Axial contrast-enhanced portal venous phase CT images obtained in the same patient at baseline (**A**, T0) and follow-up (**B**, T1), approximately 12 months apart. Total skeletal muscle segmentation is shown in red and includes the bilateral psoas, paraspinal muscles, quadratus lumborum, rectus abdominis, transversus abdominis, and internal and external oblique muscles. The bilateral psoas muscles are highlighted in blue and were used for attenuation measurements, with regions of interest positioned within the muscle belly while avoiding fascia, vessels, focal calcifications, and macroscopic intramuscular fat. Adipose tissue is highlighted in yellow; subcutaneous adipose tissue is clearly delineated between the skin and the external abdominal wall musculature, whereas visceral adipose tissue is defined within the abdominal muscular compartment. Segmentation thresholds were −29 to +150 HU for skeletal muscle, −190 to −30 HU for subcutaneous adipose tissue, and −150 to −50 HU for visceral adipose tissue. Quantitative body-composition parameters are reported below each panel. CT, computed tomography; HU, Hounsfield units; T0, baseline; T1, follow-up.

#### 3.3.1 Exploratory sensitivity analysis according to metastatic status

To explore whether baseline metastatic status materially influenced the primary longitudinal finding, percentage SMI change was compared between patients with and without metastatic disease. Median SMI change was −7.1% [IQR −8.0 to −4.7] in patients with metastatic disease and −6.6% [IQR −8.0 to −2.9] in patients without metastatic disease. The between-group difference was not statistically significant (Mann–Whitney p=0.288). The direction of SMI change was therefore similar in both groups. Given the limited subgroup sizes, this analysis was considered exploratory and was not interpreted as evidence of effect modification by metastatic status.

### 3.4 Relationship between BMI and CT-detected muscle loss

The percentage change in BMI showed only a weak relationship with the percentage change in SMI (Spearman ρ = 0.24, p=0.14). Several patients experienced substantial skeletal muscle loss despite stable or increasing BMI.

Among the 23 patients with an SMI reduction ≥5%, 15 (65.2%) had a stable or increased BMI during the same interval. Conversely, BMI change alone did not distinguish patients with SMI decline ≥5% from those with a smaller decline (**Figure 4**).

**Figure 4:**
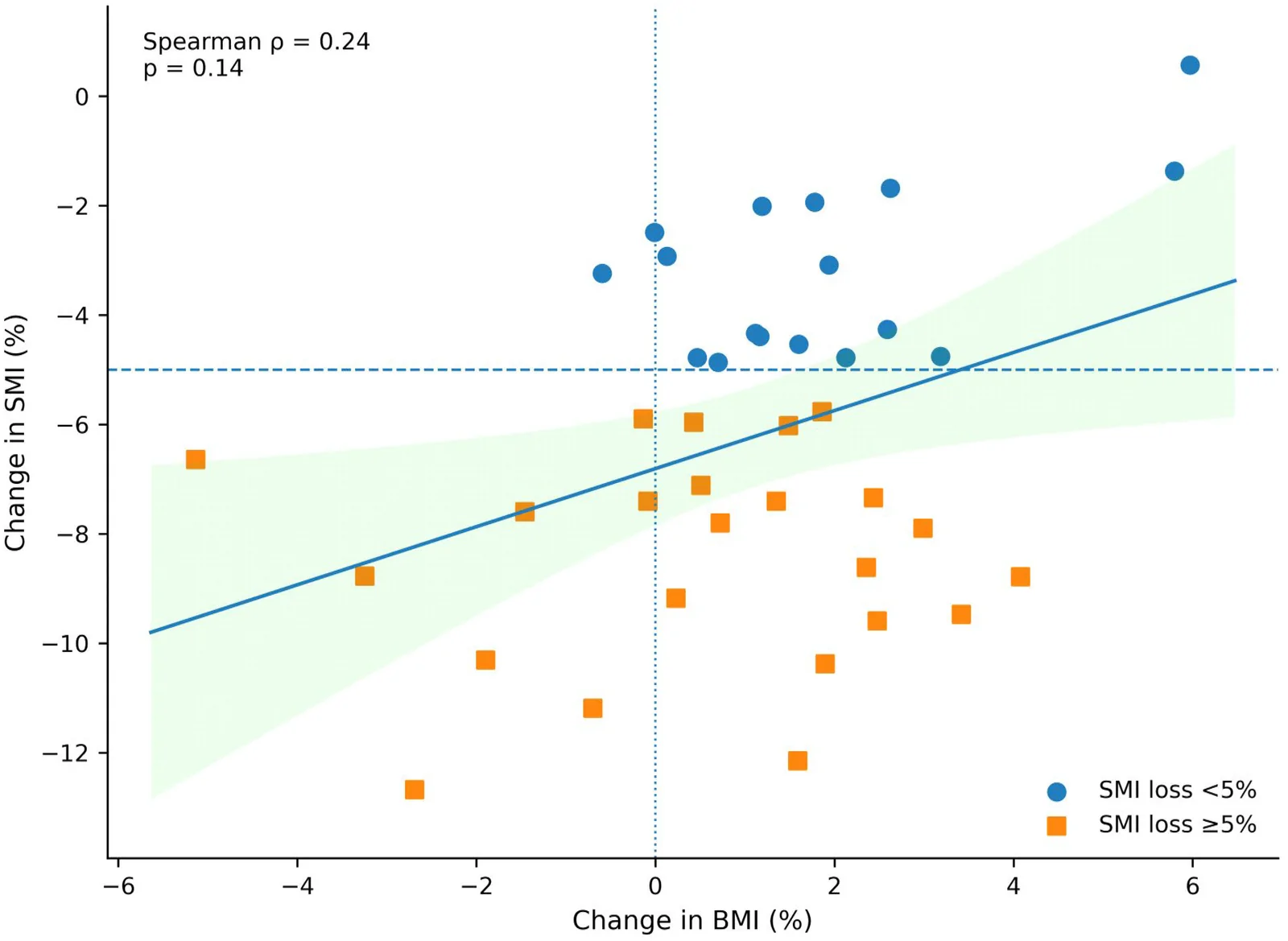
Relationship between longitudinal changes in body mass index and skeletal muscle index. Scatterplot showing percentage changes in BMI and SMI between T0 and T1. The weak correlation between the two measures illustrates that conventional weight-based assessment incompletely reflects CT-detected skeletal muscle remodeling during androgen deprivation therapy. BMI, body mass index; SMI, skeletal muscle index.

### 3.5 Exploratory factors associated with SMI decline ≥5%

Patients were classified according to the prespecified exploratory threshold of SMI decline ≥5%. Twenty-three patients (57.5%) met this threshold. Patients with SMI decline ≥5% had lower baseline psoas attenuation than those with SMI decline <5% (38.9 ± 5.3 vs. 43.8 ± 5.6 HU; p=0.007). Descriptive differences were also observed in age and metastatic status.

Given the limited cohort size, a prespecified parsimonious logistic regression model including age, baseline psoas attenuation, and metastatic status was performed.

In the exploratory three-variable model, lower baseline psoas attenuation was associated with SMI decline ≥5% (OR 1.14 per 1-HU decrease; 95% CI 1.02–1.28; p=0.024). Age (OR 1.06 per year; 95% CI 0.96–1.18; p=0.25) and metastatic disease (OR 1.82; 95% CI 0.46–7.16; p=0.39) were not statistically associated with the outcome. Given the limited sample size, these estimates should be interpreted as exploratory associations rather than as evidence of independent predictive performance (**Table 3**).

**Table 3:** Exploratory logistic regression analysis of factors associated with SMI decline ≥5%.

Variable	Adjusted OR	95% CI	p-value
Age, per 1-year increase	1.06	0.96–1.18	0.25
Baseline psoas attenuation per 1-HU decrease	**1.14**	**1.02–1.28**	**0.024**
Metastatic disease	1.82	0.46–7.16	0.39

Given the limited sample size and number of events, estimates are exploratory and should not be interpreted as establishing independent predictive effects. Bold values indicate statistical significance at p<0.05.

### 3.6 Exploratory post-T1 outcome analysis

After the T1 landmark examination, patients were followed for a median of 18.3 months [IQR 12.7–25.1]. Five deaths occurred during follow-up. Because of the very limited number of events, the survival analysis was considered hypothesis-generating and is presented in **Supplementary Figure S1**. No reliable inference regarding the prognostic significance of SMI decline can be drawn from this analysis.

### 3.7 Reproducibility

Interobserver reproducibility was high for all CT-derived measurements. ICC values were 0.97 for SMI, 0.94 for psoas attenuation, 0.98 for SATI, 0.97 for VATI, and 0.93 for vertebral trabecular attenuation.

## 4 Discussion

In this longitudinal cohort of men with prostate cancer, routine oncologic CT demonstrated concurrent changes in several body-composition compartments during approximately 12 months of follow-up after ADT initiation. SMI and psoas attenuation decreased, SATI and VATI increased, and vertebral trabecular attenuation declined, whereas BMI remained largely unchanged.

The principal contribution of the study is therefore not the isolated observation of muscle loss or fat gain, which has been reported previously, but the simultaneous assessment of muscle quantity, muscle attenuation, abdominal adiposity, and vertebral trabecular attenuation within the same routinely acquired CT examinations. Its inclusion was intended to capture a complementary skeletal dimension rather than to derive a diagnostic bone-density measure.

The reduction in SMI observed in our cohort is consistent with previous longitudinal studies of men receiving ADT (6, 8, 9, 11, 20). Importantly, BMI changed only modestly and correlated weakly with SMI change. Fifteen of the 23 patients with SMI decline ≥5% had stable or increased BMI, illustrating that conventional weight-based measures may incompletely capture changes in tissue composition. Psoas attenuation also decreased, suggesting that longitudinal changes involved both muscle quantity and radiodensity. Low skeletal muscle attenuation and visceral adiposity have also been associated with adverse outcomes in other oncologic populations (21). CT-derived muscle attenuation and SMI should nevertheless be regarded as complementary structural measures rather than diagnostic criteria for sarcopenia, which requires assessment of muscle strength and physical performance (22).

Adipose and skeletal measurements changed concurrently with the muscle parameters. SATI increased more markedly than VATI, while vertebral trabecular attenuation declined during follow-up. The inclusion of vertebral attenuation extends previous CT body-composition studies by incorporating a skeletal measure within the same longitudinal imaging framework. Because the measurements were obtained from contrast-enhanced portal venous phase CT, vertebral HU were interpreted only as an opportunistic marker of longitudinal trabecular attenuation rather than as an estimate of DXA-equivalent bone mineral density or a diagnostic measure of osteoporosis.

Lower baseline psoas attenuation was associated with SMI decline ≥5% in an exploratory three-variable logistic model. This finding should be interpreted cautiously. Muscle attenuation and SMI reflect different CT-derived characteristics of muscle composition and quantity, respectively, but derive from the same anatomical compartment and may contain partially overlapping biological and measurement information. Moreover, the limited cohort size prevented comprehensive adjustment for treatment exposure, baseline body composition, comorbidities, nutritional status, and other potential confounders. The observed association should therefore be considered hypothesis-generating rather than evidence that baseline psoas attenuation is an independent predictive biomarker.

The findings also support the feasibility of extracting multidimensional quantitative information from CT examinations already obtained for oncologic care without additional image acquisition or radiation exposure. However, the present study does not establish that CT-derived muscle–fat–bone phenotyping improves clinical outcomes or should trigger specific interventions. Rather, it demonstrates the feasibility of longitudinal opportunistic assessment and provides a rationale for prospective studies evaluating whether these measurements add clinically useful information beyond conventional oncologic and anthropometric variables.

Several limitations should be emphasized. First, the retrospective, single-center design and small cohort limit statistical power and generalizability. Most importantly, the absence of an untreated or matched comparator group precludes attribution of the observed changes specifically to ADT. Aging, disease progression, metastatic burden, concomitant systemic or radiation therapies, nutritional status, physical activity, comorbidities, and other unmeasured factors may have contributed. Because treatment regimens were heterogeneous and often combined, the present sample was not sufficiently large to disentangle the independent contribution of individual ADT classes, androgen-receptor pathway inhibitors, chemotherapy, and radiotherapy to the observed tissue changes. Baseline comorbidities, including hypertension, diabetes, cardiovascular disease, and chronic kidney disease, may also influence muscle composition, metabolic status, and physical activity; their independent effects could not be reliably modeled and therefore remain a potential source of residual confounding. The limited sample size also reduces the precision of subgroup and multivariable estimates and precludes definitive assessment of effect modification by disease stage or treatment regimen. Accordingly, the observed longitudinal pattern should not be assumed to apply uniformly across all prostate cancer populations, disease stages, or treatment regimens. Selection bias is possible because inclusion required both an eligible baseline CT and a technically comparable follow-up examination, and patients who died before T1 could not contribute to the paired imaging analysis. Residual variability in contrast administration and acquisition parameters may have influenced attenuation measurements despite technical matching. Functional measures of muscle strength, physical performance, dietary intake, testosterone levels, and DXA measurements were not systematically available. Although interobserver reproducibility was high, intra-observer reproducibility was not separately assessed and should be evaluated in future validation studies. Finally, only five deaths occurred after the T1 landmark; consequently, the supplementary survival analysis is strictly hypothesis-generating and cannot establish prognostic significance. Larger prospective, preferably multicenter and controlled studies are required to confirm these longitudinal patterns and determine their clinical relevance.

## 5 Conclusion

Routine oncologic CT demonstrated concurrent longitudinal changes in skeletal muscle quantity and attenuation, abdominal adiposity, and vertebral trabecular attenuation following ADT initiation, whereas BMI changed only modestly. These findings support the feasibility of integrated opportunistic muscle–fat–bone assessment using routinely acquired CT examinations without additional imaging acquisition. Because of the retrospective single-center design, limited sample size, clinical heterogeneity, and absence of a comparator group, the observed changes cannot be attributed specifically to ADT and their prognostic or clinical utility remains to be established. Larger prospective and controlled studies are required to validate these findings and determine whether longitudinal CT-derived phenotyping provides clinically useful information beyond conventional assessment.

## Data Availability

The datasets presented in this article are not readily available because they contain clinical and imaging data subject to patient-confidentiality, ethical, and institutional restrictions. Raw data are therefore not publicly available or routinely shared. Any access would require specific institutional authorization and compliance with applicable data-protection requirements. Requests to access the datasets should be directed to giuseppe.guglielmi@unifg.it.

## Supplementary material

Supplementary Figure 1 Exploratory Kaplan–Meier analysis according to longitudinal SMI decline. Survival was calculated from the T1 landmark CT examination, at which longitudinal SMI change could first be determined. The analysis is presented for descriptive, hypothesis-generating purposes only because the limited number of events precludes reliable prognostic inference. SMI, skeletal muscle index.

Supplementary Figure 2 Individual longitudinal changes in CT-derived body-composition parameters. Paired T0-to-T1 trajectories are shown for **(A)** skeletal muscle index, **(B)** mean psoas attenuation, **(C)** subcutaneous adipose tissue index, **(D)** visceral adipose tissue index, and **(E)** vertebral trabecular attenuation. Each line represents one patient; cohort mean ± standard deviation is superimposed at each time point.
