# Supplementary material for: Longitudinal CT-derived muscle–fat–bone remodeling following androgen deprivation therapy initiation in prostate cancer
Source: Front Urol. 2026 Oct 8;6:1985221. doi: 10.3389/fruro.2026.1985221 (PMC13686873; doi:10.3389/fruro.2026.1985221)
Supplement: Supplementary file 1 [file Supplementaryfile1.pdf]

Supplementary Material

Longitudinal CT-Derived Muscle–Fat–Bone Remodeling Following Androgen Deprivation Therapy Initiation in Prostate Cancer

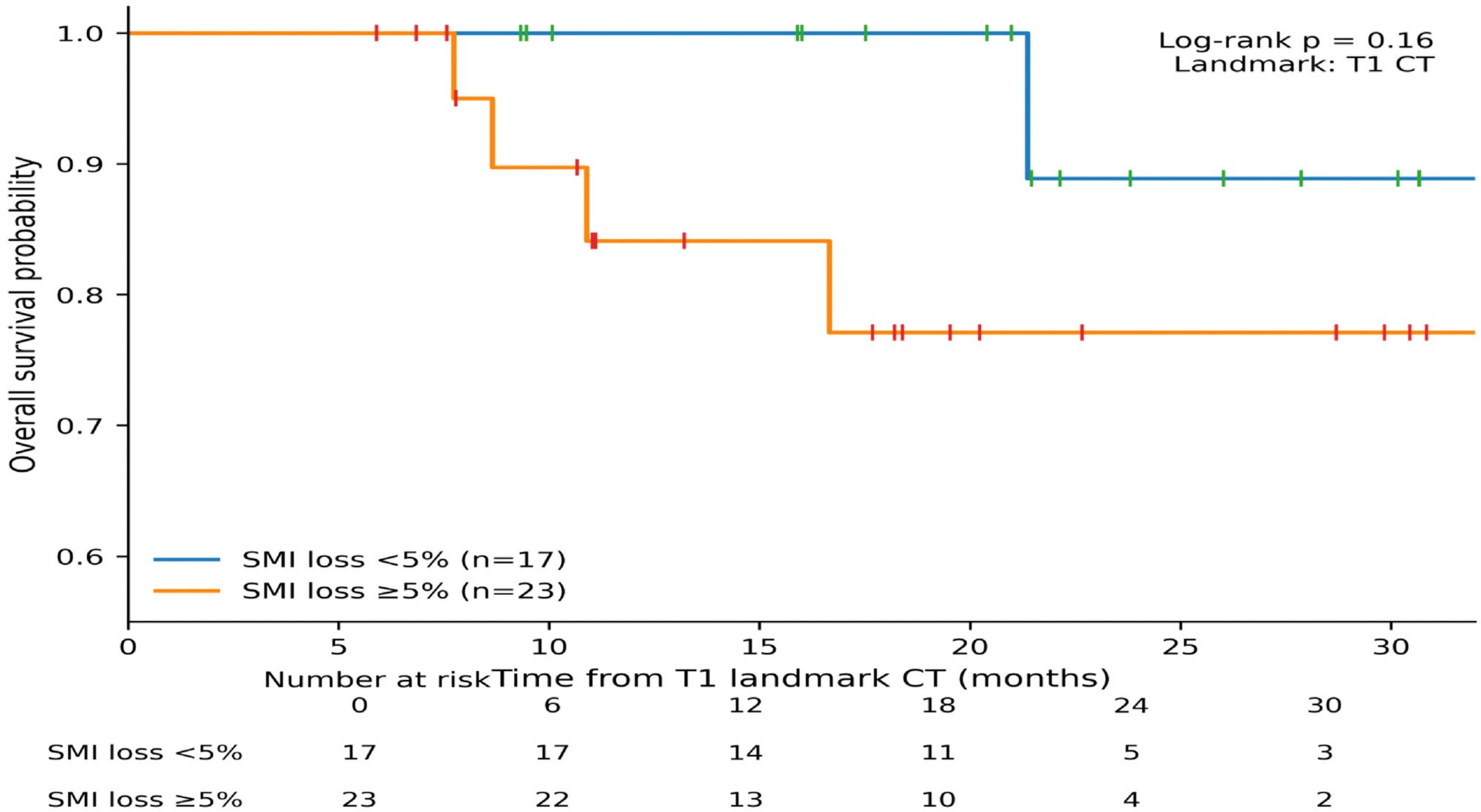

Supplementary Figure S1. Exploratory Kaplan–Meier analysis according to longitudinal SMI decline. Survival was calculated from the T1 landmark CT examination, at which longitudinal SMI change could first be determined. The analysis is presented for descriptive, hypothesis-generating purposes only because the limited number of events precludes reliable prognostic inference. SMI, skeletal muscle index.

Supplementary Figure S2 (panels A-B)

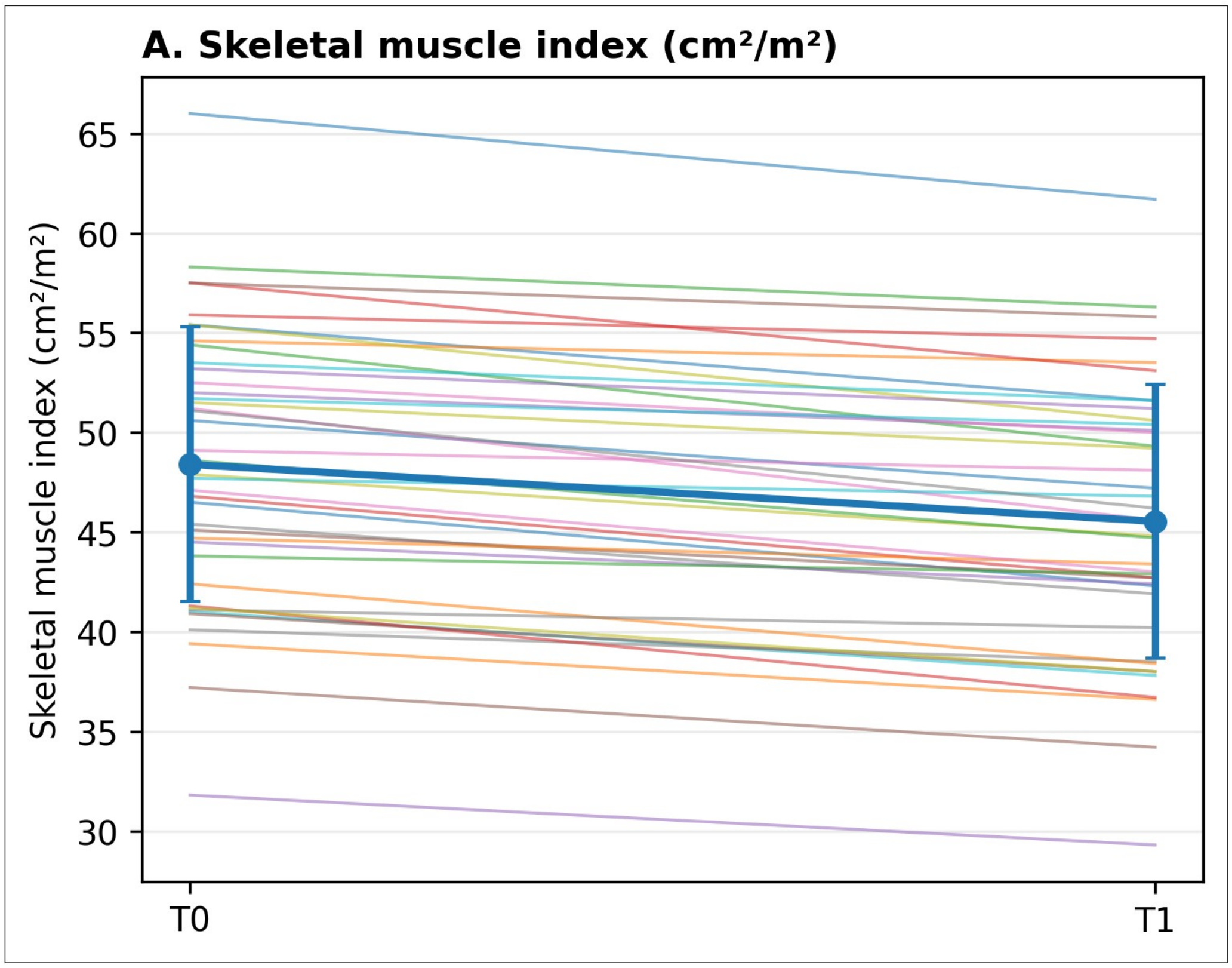

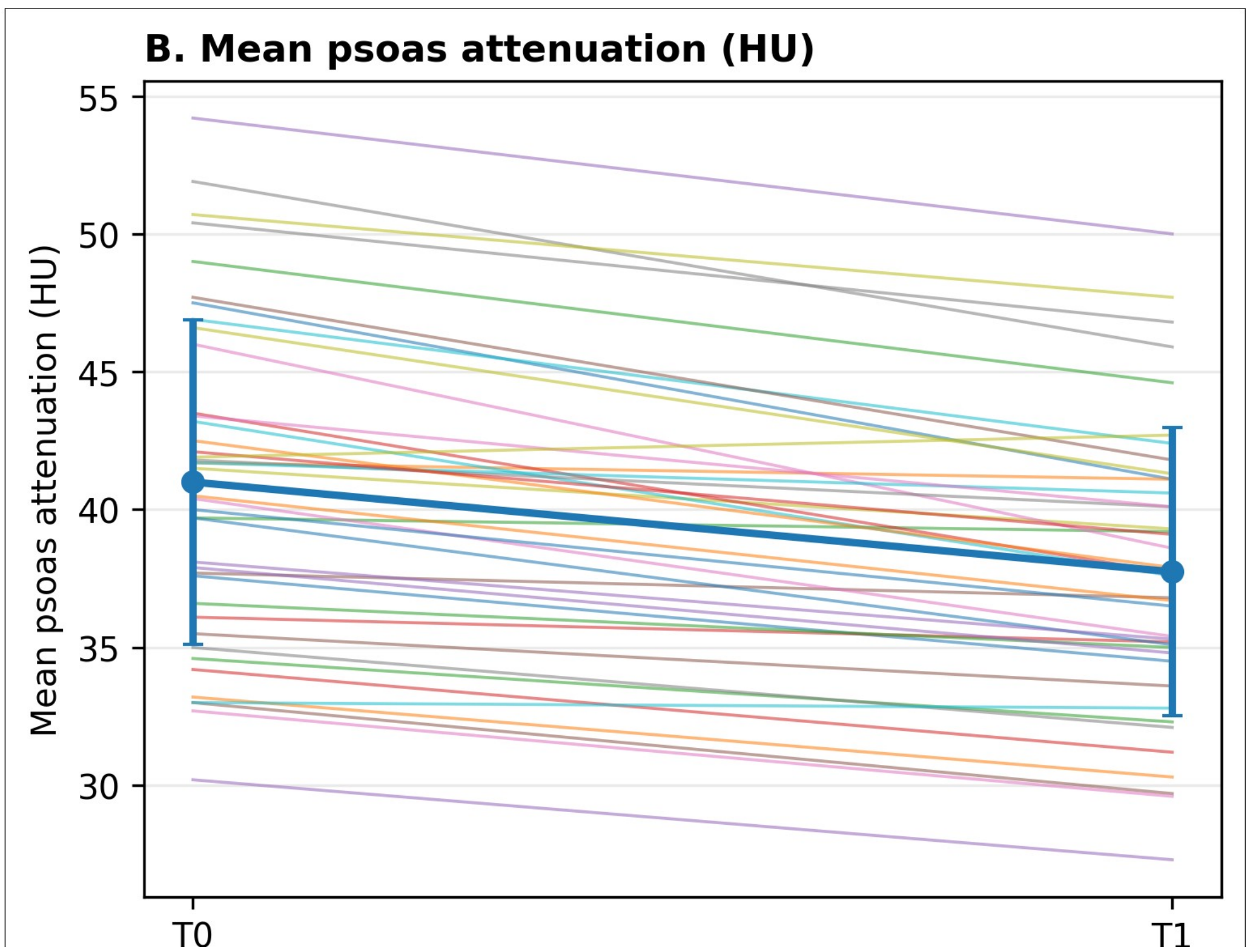

Supplementary Figure S2 (panels C-E)

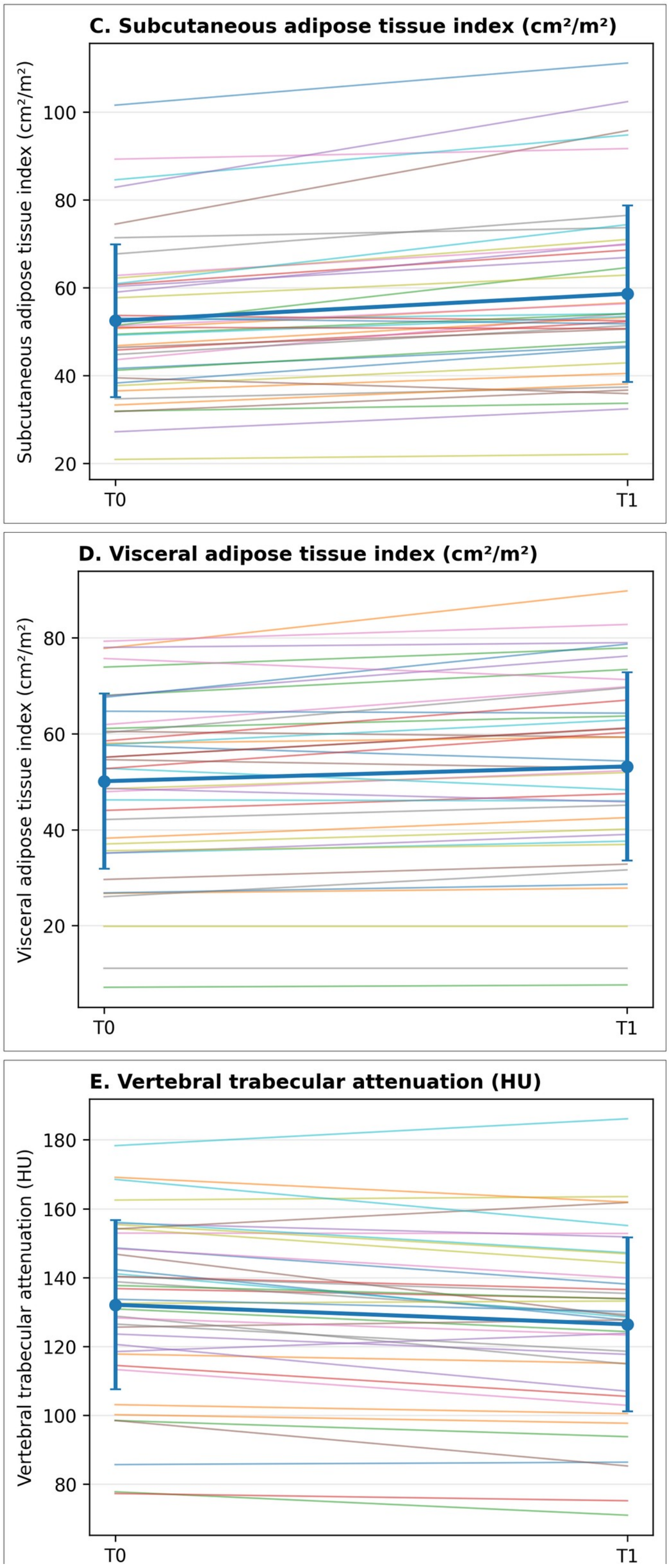

Supplementary Figure S2. Individual longitudinal changes in CT-derived body-composition parameters. Paired T0-to-T1 trajectories are shown for (A) skeletal muscle index, (B) mean psoas attenuation, (C) subcutaneous adipose tissue index, (D) visceral adipose tissue index, and (E) vertebral trabecular attenuation. Each line represents one patient; cohort mean ± standard deviation is superimposed at each time point.
